# The Goodyear Company's Patent

**Published:** 1879-06

**Authors:** 


### ARTICLE VI.

### *The Goodyear Company's Patent.*

(An Explanation in Behalf of the Dentists.)

To the Editor of the New-York Times:

The statement made in your paper of 4th inst. in the article signed "Goodyear Dental Vulcanite Company, by E. Ernst Caduc, Secretary," and also those made by "M. D." in your issue of April 30th, are so incorrect and so unfair that I send you page 169 of the "Contemporary Biography of New-York," and I ask you, in simple justice to the dental profession in this country, to publish the portion relating to Dr. Thomas Bryan Gunning's legal contest with, and complete defeat of, Josiah Bacon and his employers. J. A. B.

"The World's Fair, held in London in 1851, contained several exhibits of artificial teeth set on plastic basis or plates, but nothing approaching to the hard rubber, for which an American patent was granted to Nelson Goodyear in May of the same year. His brother Charles obtained his first idea of applying it to artificial teeth while in Europe to attend the fair, and experiments were subsequently made to adapt it for use in the mouth, but with no useful result until 1857, and this of little importance for two or three years later. In 1861 Dr. Gunning purchased the right to use the Goodyear patents through all their extensions. In June, 1864, the patents had less than a year to run, but a new patent for the application of the rubber to artificial teeth was obtained on the ground that one John A. Cummings had perfected the process in 1855. This patent, having a 17 year term, extended the monopoly from 14 years to 30, that is, to 1881, and to enforce this, the Goodyear patents being extended seven years, a license was given to use both sets of patents, so that the Goodyear patents sustained the Cummings. This was resisted by many who admitted the validity of the Goodyear patents, but suits were brought, and the Cummings patent sustained by the United States Circuit Court in Boston, in 1866. As Dr. Gunning would not be made an example of submission to what he deemed an illegal claim, several bills in equity were filed against him, but the company rested as soon as his answers were filed. In the Autumn of 1871 the case known as the 'Gardner appeal' was presented to the dentists, who rallied to its support, as urgently advised by leading members of the profession. But the United States Supreme Court decided adversely to the dentists, and affirmed the Cummings patent on May 6th, 1872, the day the Goodyear patents expired. The company then commenced another (the fourth) suit against Dr. Gunning, who, finding that his patent council had accepted a retainer from his opponents and could not appear for him, instructed his attorney to do so instead. Gunning's desire was to have the decision in the Gardner case recalled, and he offered to break the Cummings patent if the American Dental Association would co-operate with him. As this offer was not accepted, it was impossible to form a combination to bring the company to a fair fight on the merits of the case. Dr. Gunning, therefore, decided to force the company to a settlement with himself. The result was, that rather than have the answer which he had dictated filed, they acknowledged his right to do as he had done, and stipulated that he should use all the improvements claimed under their patents without molestation in the future. Their bill was dismissed by Judge Woodruff, October 18th, 1872, the company paying the taxed costs. Dr. Gunning had warned the dentists against the Gardner appeal case. Now, although his personal interest in the matter was at an end, he still earnestly desired to release them from unjust exaction. Having submitted the matter to Charles O'Conor, Dr. Gunning sought Judge Black, and gave him full explanations, together with Mr. O'Conor's written views. Judge Black then determined to make the motion before the Supreme Court, and it being shown that the counsel on both sides of the Gardner case had been paid by the complainant, on March 3rd, 1873, the court dismissed that case and recalled its mandate, although it had issued to the Circuit Court. This is the first instance in which the Supreme Court of the United States ever revoked its decision. By this revocation the dentists were again at liberty to contest the Cummings patent."

---

